# From Participants to Community Partners: A Novel Community-Based Participatory Research (CBPR) Approach to Autistic-Led Inquiry in Digital and Virtual Environments

**DOI:** 10.3390/healthcare14060702

**Published:** 2026-03-10

**Authors:** Vivian Darlene Grillo, Margherita Zani, Vittoria Veronesi, Paola Venuti

**Affiliations:** 1 Observation Diagnosis and Training Laboratory (ODFLab), 38068 Rovereto, Italy; viviandarlene.grillo@unitn.it (V.D.G.); margozani@gmail.com (M.Z.); vittoriaveronesi23@gmail.com (V.V.); 2 Department of Psychology and Cognitive Sciences, University of Trento, 38068 Trento, Italy; 3 Fondazione Bruno Kessler, 38123 Trento, Italy

**Keywords:** autism, neurodiversity-affirming research, virtual reality, social virtual environments, VRChat, participatory methods, reflexive thematic analysis, artificial intelligence integration

## Abstract

**Background/Objectives**: Autism research has often interpreted autistic sociality through neurotypical norms, limiting ecological accounts of autistic meaning-making and context-sensitive support needs. Social virtual environments (SVEs), such as VRChat, allow modulation of sensory exposure, social distance, and participation pace, potentially enabling autistic-led interaction with greater autonomy and predictability. This study examined how autistic young adults co-construct meanings around social interaction, identity, and self-regulation in peer-led discussions within an SVE; identified context-sensitive processes relevant to well-being; and evaluated the feasibility and acceptability of SVEs as a participatory research setting. **Methods**: Sixteen autistic young adults (18–38 years; DSM-5-TR, Level 1) participated in nine remote sessions conducted in VRChat, coordinated via a co-designed Discord server. The peer-led discussions were audio-video recorded, transcribed, and anonymized. Data were analyzed using reflexive thematic analysis, combining inductive session-level coding, cross-session thematic clustering, and participatory refinement with community partners. **Results**: Autistic experience was framed as a context-dependent negotiation of interpretive risk, interactional workload, masking-related energy costs, and epistemic injustice, alongside future-oriented accounts emphasizing access, dignity, and systemic redesign. Observational memos documented multimodal participation, distributed peer facilitation, and accessibility-relevant sensitivities to environmental stability. Community partners reported positive experiences and supported the acceptability of private-world VRChat sessions. **Conclusions**: Peer-led discussions in an SVE can support ecologically grounded, participant-centered qualitative research, offering methodological opportunities to study autistic meaning-making under conditions that reduce demands and risks.

## 1. Introduction

### 1.1. Autism Research Frameworks

Autism research has long been dominated by deficit-oriented frameworks that interpret autistic social behavior and communication primarily through neurotypical (NT) norms and expectations [1,2,3,4]. Within this tradition, autistic interaction styles are framed as impairments or deviations that require correction, rather than as valid forms of human variation [1,2,3,4]. These assumptions have shaped not only how autistic behavior is interpreted, but also which research questions are prioritized, which outcomes are considered meaningful, and whose interpretations are treated as authoritative [5,6,7,8]. As a result, autistic perspectives have frequently been marginalized within autism science itself [3,9,10].

In response, the neurodiversity movement and critical autism scholarship argue that autism should be understood as a naturally occurring form of neurocognitive diversity (not as a disorder defined solely by deficits) and advocate for shifting autism research toward approaches that redistribute interpretive authority, foreground autistic priorities, and build accounts of autistic experience that are not filtered exclusively through NT norms [11,12].

In autism research, social challenges have often been treated as intrinsic limitations of the autistic individual (e.g., Theory of Mind [13]; Social Motivation Theory [14]; gaze avoidance as deficit rather than regulation [15]). However, growing evidence suggests that social interaction outcomes are directly tied to the neuro-types involved (e.g., the Double Empathy framework [16]; social challenges as relational and context-dependent [17]; the assumption that autistic people lack social motivation is challenged [18]) and depend on contextual factors and constraints (e.g., sensory load, uncertainty, interactional norms and expectations, and regulatory demands) [17,19,20]. For autistic people, environments characterized by unpredictability, intense sensory stimulation, and implicit social rules can substantially increase cognitive and emotional strain [19,21,22], whereas contexts that allow greater control and predictability may support more effective and comfortable social participation [23,24]. 

This mismatch between autistic people and NT-designed contexts (relational and environmental) has significant consequences. It is particularly pressing in light of the substantial mental health burden [25] and reduced quality-of-life outcomes reported among autistic adults [26,27,28,29], alongside limited evidence about the protective processes that sustain well-being (e.g., forms of social connection, self-regulation strategies, and environments that reduce chronic stress) [30].

Recent syntheses report elevated rates of anxiety and depression in autistic adults [31]. Meta-analytic evidence indicates markedly higher prevalence of suicidal ideation and suicide attempts compared with non-autistic populations, underscoring an urgent public health need for interventions and contexts that support autistic well-being rather than intensify pressure toward compliance or camouflage [32]. Camouflaging (like ‘masking’) refers to the suppression or alteration of one’s characteristics and/or behaviors to fit into a social environment that is threatening, or perceived as threatening, or non-accommodating [32].

Importantly, emerging evidence suggests that distress is not necessarily attributable to autistic traits themselves; rather, it often appears to arise from chronic exposure to social stigma, misunderstanding, sensory overload, and the sustained effort required to ‘fit’ into NT-designed environments (e.g., suppressing repetitive or self-regulatory behaviors, also known as ‘stimming’) [17,33,34,35]. The distinction between trait expression and trait suppression therefore has significant clinical and ethical implications [15,36,37]: behaviors often classified as “symptoms” (e.g., stimming or focused interests) function as stabilizing resources in contexts that permit them, while becoming sources of risk and/or harm in contexts that stigmatize or penalize them [38,39,40,41].

### 1.2. Social Virtual Environments as Predictable Contexts for Authentic Autistic Sociality

Within this broader landscape, immersive social virtual environments (SVEs) offer a promising setting for autism research. Social VR platforms such as VRChat (https://hello.vrchat.com/) enable real-time interaction through embodied avatars while allowing users to modulate key contextual aspects [24,39,40,42], including the following:

- *Self-presentation* [39,42] (i.e., personal avatar selection and/or creation based on real-life features, fictional characters, or any other imaginable preference);
- *Immersive-embodiment level* (i.e., choosing desktop-play or head-mounted display [40,43,44]);
- *Familiar location* [21,22] (i.e., engaging from the commodity and predictability of one’s own home);
- *Sensory intensity and configurable sensory exposure* (i.e., quality and quantity of audio output, in terms of sounds, music, other users’ voices, and volumes; environments with specific colors and/or backdrops, like with more or less lighting);
- *Adjustable interpersonal/social distance* (i.e., filtering who can interact with your avatar and/or selecting how far/close other users need to be in order to interact with you);
- *Communication channel* (i.e., choosing via microphone/voice, via chat/text, via avatar gestures or emotes);
- *Pacing* (i.e., ability to join or leave interactions at will; take breaks by disconnecting; and regulate the duration of engagement).

These features offer control and predictability [39,42], which may reduce some of the environmental, interactional and sensory pressures or costs of in-person sociality, while preserving important elements of in-real-life social interaction and enabling agency [17,23,39,40,42]. In other words, SVEs can function as ‘social laboratories’ that are ecologically meaningful: they preserve many real-world interaction demands (coordination, conversation, and group dynamics) while allowing users to choose and/or change the conditions under which social cognition unfolds (e.g., sensory intensity, embodiment choices, and predictability) [39,42]. This makes SVEs potentially well suited to studying autistic sociality beyond deficit framings – by investigating how social participation changes when sensory load and masking demands are reduced.

However, the scientific value of SVEs depends on *how* data are generated, collected, and interpreted. If research simply transposes NT assumptions into a novel medium, existing epistemic biases may persist. The present study therefore adopts an autism-informed and neurodiversity-affirming orientation, treating autistic communication, behavior, and social interactions as natural forms of human variation rather than deficits to be remediated.

### 1.3. The Present Study’s Positionality

The present study is part of a broader research project and is situated within a growing body of autism research on autistic adults [45]. The design and facilitation of the VRChat group sessions (see Section 2) were structured on the basis of findings from earlier phases of this project [39] and from prior qualitative work with mixed (neurodivergent and neurotypical) autism professionals [42]. In addition, the study is informed by emerging qualitative research examining autistic experiences in virtual and technology-mediated social environments, which has highlighted the role of environmental predictability, sensory modulation, and perceived safety in shaping social participation [22,40,46].

In fact, this study was intentionally developed to minimize unnecessary demands and support participant agency. Several features were implemented to reduce *(a) social pressure* and *(b) cognitive load*, while promoting *(c) predictability* and *(d) voluntary participation*. For example, to reduce social pressure, participants were introduced to the group context gradually over six months, beginning with one-to-one individual meetings with the first author before moving to a small group and, finally, the larger, discussion group. Participants also presented as avatars of their choice. To reduce cognitive load, each participant received illustrated Discord and VRChat user manuals that they could consult at any time. Predictability was supported through a consistent session structure (i.e., collaboratively selecting a virtual world and exploring it together, followed by an open discussion of a previously proposed topic). Participation was voluntary and flexible, with a ‘drop-in’ format that allowed participants to connect if, when, and for as long as they wished. These design choices reflect principles associated with neurodiversity-affirming, autism- and trauma-informed practices [47,48] aimed at reducing interpersonal threat and facilitating engagement [49,50,51].

While much of the existing virtual reality literature in autism has framed immersive environments primarily as tools for social skills training or behavioral remediation [52,53,54], recent reviews have highlighted both the limited generalizability of such interventions and the ethical concerns related to masking and normative social performance demands [37,55,56]. The present study diverges from this traditional intervention paradigm by conceptualizing SVEs not as training tools, but as alternative social ecologies in which autistic interactional practices and meaning-making can be examined on their own terms [40]. Instead of evaluating skill acquisition or behavioral change, the study examines how autistic young adults experience, interpret, and negotiate social interaction when environmental constraints are reconfigured.

The approach adopted throughout this study is consistent with recommendations emerging from participatory autism research [57,58], which suggest that accounts of autistic experience are more accurate and ecologically valid when autistic individuals are positioned as active contributors to knowledge production (‘epistemic partners’) rather than as passive ‘subjects’ [3,11,59,60,61,62]. While prior participatory and human–computer interaction studies have primarily focused on technology acceptance, usability, and design affordances [46,63,64], the present study complements this work by shifting analytic attention to collective, peer-led meaning-making processes as they unfold in situ within an immersive social platform.

Methodologically, this contribution builds on emancipatory disability scholarship and Community-Based Participatory Research (CBPR) approaches that emphasize shared decision-making and co-ownership of knowledge [61,65,66,67]. This approach is particularly appropriate for domains such as social interaction, emotion regulation, masking, and sensory experience, which are areas that have historically been interpreted through non-autistic, normative-based social frameworks [36,68]. To address this limitation in prior research, the present study employs peer-led, participant-prioritized group discussions conducted in VRChat, coordinated through community-based digital practices. This methodological design enables participants not only to set the agenda of discussion but also to collectively negotiate, validate, and differentiate meanings across heterogeneous autistic experiences.

Therefore, the primary aim of the present study was to ecologically examine how autistic young adults co-construct or make sense of social interaction, autistic identity, and self-regulation when they participate in peer-led group discussions within an immersive SVE. Specifically, the study seeks to (1) document how autistic young adults collectively articulate social challenges and resources, under conditions of increased autonomy and predictability; (2) identify context-sensitive processes that may function as protective factors for well-being; and (3) evaluate the feasibility and acceptability of SVEs as participatory, neurodiversity-affirming research settings. 

## 2. Materials and Methods

### 2.1. Study Design and Methodological Orientation

This naturalistic qualitative study [69] used a community-informed, participant-led [70] longitudinal design to explore autistic young adults’ priorities and meaning-making within digital and virtual social environments. The methodological framework was constructivist (knowledge as co-constructed through interaction and context) [71,72,73,74]. In other words, the research team acted like a ‘fly on the wall’ and listened to what autistic young adults cared to share, while trying to understand how social cognition and emotional life unfold when environments are redesigned to reduce interpretive risk (neurodivergent-neurodivergent interactions, or ND-ND) and increase agency (i.e., peer-led) – an approach with direct implications for intervention design, digital mental health, and future, inclusive neurotechnology.

Because autistic experiences are often misinterpreted through external frameworks and undervalued as knowledge, the study was also informed by CBPR [75] and by scholarship on epistemic injustice in disability/autism research [35]. This orientation shaped both the data-generation setting and structure and the interpretive process of the data.

Data generation was structured within a research context that held the following design features: (a) clear *participation conditions*, including voluntary session attendance and home-based access; (b) an *interaction format* consisting of peer-led group discussions facilitated by the autistic participants themselves; (c) *familiar digital practices*, with sessions coordinated through a dedicated Discord server (https://discord.com/; headquarter in San Francisco, CA, USA) used for scheduling, asynchronous communication, and session follow-up, and participation occurring via desktop mode rather than head-mounted displays; (d) *multiple communication modalities*, including voice, text chat, and avatar-mediated gestures or emotes, which participants could select based on preference, energy level, sensory load, or situational needs; (e) *self-regulation affordances*, such as the ability to mute audio, take breaks, observe (“lurk” [39]) before contributing to the discussion, or temporarily disengage as needed; (f) *socio-technical features of VRChat* (hello.vrchat.com; San Francisco, CA, USA), including avatar-mediated interaction and explicit turn-taking cues, which supported flexible participation and reduced interactional demands. 

The interpretive process was guided by the same orientation; therefore, following the initial analytic phase, autistic community partners were invited to review preliminary themes, assess their resonance and accuracy, and confirm, challenge, or refine interpretations. Their input informed theme consolidation and the development of analytic implications discussed in the Discussion, thereby enhancing analytic rigor while redistributing interpretive authority within the research process.

### 2.2. Ethics, Safeguarding Privacy, and Trauma-Informed Practices

The study was approved (protocol code: Progetto Grillo; CE registry number: A978) by the Ethics Committee of the Autonomous Province of Trento’s local health authority (Azienda Provinciale per i Servizi Sanitari di Trento, APSS) on 20 June 2024, Italy. Informed consent was obtained prior to participation, and consent was treated as ongoing, with reminders that participants could pause, take breaks, leave, or withdraw at any time without consequences.

Throughout the study, (a) predictable structures and transparent communication, (b) participant control over engagement, and (c) minimizing exposure-related stressors were prioritized. A licensed psychologist was always on call and available during sessions for support in case of distress, and the research team monitored for signs of discomfort. No adverse events requiring clinical intervention occurred.

### 2.3. Digital–Virtual Context and Setting

Data were generated across nine remote sessions conducted between April and May 2025. Sessions took place in VRChat (desktop mode; no head-mounted displays to minimize potential side effects [76]), with preparatory coordination and ongoing community support hosted on a Discord server co-designed with autistic participants in a prior phase of the project.

VRChat enabled participants to interact via customizable avatars in participant-selected worlds while remaining in self-managed physical environments (typically the home), which participants reported as reducing sensory and social stressors and aligning with everyday digital communication practices.

This setting was selected to maximize ecological validity and reduce researcher-induced reactivity: rather than an institutional laboratory or clinical room, discussion unfolded in participants’ familiar communication ecosystems, with multimodal interaction options (voice, text, avatar-gestures, etc.).

### 2.4. Participants and Sampling

As this study constituted one component of a broader, multi-phase research project, the number of participants included was not predetermined but emerged organically based on participant availability and engagement. Participation in this phase was entirely voluntary and participant-led; consequently, the final sample reflects those individuals who were actively involved during the data collection period. Variations in participation were influenced by factors such as competing commitments, limited interest in the specific topic(s) addressed, fluctuations in energy levels and availability, and experiences of burnout [77]. Accordingly, the present study reports only on participants who actively contributed to the sessions relevant to this phase of the research, which accounts for the variable number of participants across sessions (see: Appendix A).

Participants were autistic young adults (N = 16), aged 18 and 38 years (*M* = 26.8, *SD* = 5.8), with an equal gender distribution (assigned female at birth [AFAB] = 8; assigned male at birth [AMAB] = 8). All participants had a formal Level 1 diagnosis (indicating low support needs; DSM-5-TR, *Diagnostic and Statistical Manual of Mental Disorders, Fifth Edition, Text Revision* [78]) and sufficient receptive and expressive language abilities to engage in group-based dialogue via voice and/or text-based communication. In accordance with the ethics committee requirements (protocol code: Progetto Grillo; EC registry number: A978), only formally diagnosed autistic young adults were eligible; self-diagnosed individuals were excluded. Additional inclusion criteria required that participants had no prior use of VRChat and no hands-on experience with virtual reality (VR). This was intended to minimize confounding effects of platform-specific familiarity and to examine first-time engagement under comparable conditions; specifically, desktop-play mode, which is a screen-based interaction modality that does not require a headset.

A purposive sampling strategy was employed to recruit individuals motivated to participate in a VR research project. Recruitment occurred through hospitals and autism-focused clinical services, university-affiliated support programs, clinical research laboratories, and neurodiversity advocacy networks in Italy, supplemented by targeted flyers and ad hoc informational materials. Partner organizations disseminated the study invitation ***only*** to eligible patients, clients, or contacts, thereby confirming that potential participants had received a formal diagnosis. Participation was voluntary; individuals could attend or opt out at any given moment without penalty or repercussions.

To further characterize the sample within the broader research project, individual in-depth interviews were conducted and participants completed standardized measures (i.e., Social Responsiveness Scale-2, SRS-2) administered outside the present qualitative analysis. Moreover, a questionnaire was developed and completed by the autistic volunteers to capture aspects of their digital practices, preferences, and prior experiences or relationships with online social spaces. These data were collected to describe participant characteristics and support interpretability at the project level; however, the present paper does not use questionnaire scores as an inclusion threshold or as a basis for subgroup comparison, consistent with the study’s naturalistic, exploratory, qualitative aims.

All participants were Italian speakers, residing in or originally from five countries. The sample included participants who were female- and male-assigned at birth, with heterogeneity in diagnostic timing (e.g., later diagnosis more common among female participants) and diverse background and cultural experiences, which enriched comparative reflections and collaborative meaning-making during the peer-led discussion.

### 2.5. Procedure and Participant-Led Topic Selection

The study prioritized participant agency and peer-to-peer meaning-making. Sessions were peer-led and self-facilitated, with participants selecting discussion topics on the Discord server and bringing them into VRChat sessions. This approach intentionally avoided rigid group interview or focus group schedules and researcher moderation, consistent with naturalistic inquiry aims to minimize artificial constraints and enable interactional dynamics to unfold organically.

The research team maintained a supportive, low-intrusion presence: they provided technical assistance when requested and ensured that participation logistics were predictable and accessible (e.g., session reminders, both privately on participants’ preferred platform—e.g., Discord, WhatsApp (https://www.whatsapp.com; Menlo Park, CA, USA), via personal email address—and publicly on Discord; clear instructions; and optional modalities), without directing content or discussion.

*Recording approach:* Participant self-recording was considered but excluded following an accessibility review due to increased executive-function demands (e.g., device configuration, multi-step setup, troubleshooting, time-sensitive initiation of recording, subsequent file handling/transfer to the research team). Recordings were therefore managed centrally by the research team in accordance with the approved ethical protocol, ensuring secure institutional storage and controlled access, minimization of identifying information (avatar-based capture), and standardized confidentiality and data management procedures.

### 2.6. Data Collection and Data Management

Nine VRChat ‘hangouts’ were held; eight thematic discussion sessions were audio-video recorded and stored securely in compliance with the ethics protocol. Recordings captured participants’ avatars and voice/text interaction; no physical appearance was recorded. Participants independently selected avatars and worlds, supporting privacy and comfort.

Recordings were manually transcribed and then pseudonymized (e.g., PV_01), with identifying references replaced by [ANONYMIZED] or [SENSITIVE DATA] tags. After coding and theme development, transcripts were fully anonymized and stored on encrypted, password- and two-factor-protected institutional drives. Any linkage key between participant identities and codes was securely deleted to prevent re-identification.

### 2.7. Data Analysis: Reflexive Thematic Analysis with Multi-Stage Clustering and Participatory Refinement

Nine transcripts were analyzed using an RTA approach [79,80,81,82,83,84], conducted in a sequential, multi-stage analytic process to avoid premature generalization—first prioritizing close, inductive familiarization and engagement with each discussion, then moving to cross-session synthesis via thematic clustering.

*Phase 1: Independent inductive coding (session-level).* Two coders, independently familiarized with the data, coded each session-transcript inductively, generating initial codes and session-specific candidate themes.

*Phase 2: Analytic dialogue and consensus.* Coders compared codes and candidate themes through structured discussions, using analytic memos to document convergences, tensions, and interpretive decisions, with particular attention to how meanings were negotiated within sessions. This phase aimed to enhance interpretive transparency and ensure that theme labels remained closely anchored to the dataset.

It should be noted that analytic attention focused on participants’ expressed meanings of the discussed topics at both the individual and group levels, as represented in the transcripts and analytic memos (i.e., *what* participants articulated and how meanings were negotiated across turns of talk). The analysis did not involve systematic coding of paralinguistic features such as tone, prosody, pacing, or vocal emphasis. While audio–video recordings were available, the study was designed as an exploratory analysis oriented toward meaning-making processes.

*Phase 3: Higher-order thematic clustering (cross-session integration).* Session-level themes were then treated as analytic units and examined comparatively across sessions by the first author. Clustering involved iteratively mapping thematic overlaps, contrasts, and relational links across sessions, with attention to recurrence, variation, and contextual specificity. Themes were grouped into higher-order patterns based on conceptual affinity, allowing integrative threads to be identified while preserving within-session nuance and heterogeneity. Provisional macrothemes were repeatedly reviewed against the original individual-session themes and analytic memos to ensure coherence, distinctiveness, and fidelity to participant accounts.

*Phase 4: Participatory theme refinement (autistic community partners).* Following the thematic clustering phase, participants were invited to join and to participate in collaborative meetings with the academic team. For this phase, partners were provided with accessible, scientific lay-form materials, which included the following: (a) a slide-based presentation outlining the aims of the study, the analytic process, and interim analytic decisions, and (b) a document containing the results, including theme labels, concise analytic summaries, and selected anonymised illustrative extracts.

Two participatory meetings (approximately 90 min each) were conducted online via Zoom (https://www.zoom.com/; Zoom Communications, Inc.; San Jose, CA, USA) and followed a structured yet open format. The research team presented the previously shared materials on Discord, orally, reading and explaining themes and accompanying extracts aloud to ensure accessibility and shared understanding. Each presentation segment was followed by open discussion, during which partners were invited to provide feedback without constraint or pre-specified response categories. Partners were explicitly encouraged to offer critical, corrective, or confirmatory input; to raise concerns about clarity, emphasis, or framing; to identify missing or underdeveloped experiential dimensions; and to challenge interpretations they perceived as inaccurate or shaped by neurotypical assumptions. Data was gathered in vivo by writing what was being said in real time on the shared Zoom screen. This allowed the research team to ensure that everything was reviewed by the autistic partners and correctly captured (i.e., rewording notes where necessary). Moreover, the meetings were also recorded to document feedback, interpretive suggestions, and proposed conceptual refinements. This open format also supported dialogic exchange among partners, allowing reflections to build on one another’s contributions and shared personal experience.

This “post-hoc” participatory step was intentionally proposed and scheduled several months after the sessions (data collection) to avoid influencing or shaping natural group dynamics or creating demand characteristics during data generation, while enabling substantive involvement in the interpretive and dissemination phases in line with CBPR principles. Insights from these discussions informed subsequent revisions to theme boundaries, descriptions, and analytic emphasis prior to finalization.

*Phase 5: Finalization.* Themes were finalized when they demonstrated internal coherence, distinctiveness, and were judged to provide a faithful account of the dataset and the meanings participants co-constructed.

To meet qualitative rigor standards and principles, we employed several strategies, including analytic triangulation (i.e., independent coders and consensus meetings), audit trails (documentation of all coding stages, theme decisions, and analytic memos), reflexive practice (e.g., reflections on our own positionality captured throughout analysis), attention to ecological validity (data was collected in participant-selected SVEs), thick descriptions (e.g., preservation of interactional, sensory, and contextual cues in transcripts), and transparency (detailed reporting of analytic procedures for reproducibility).

## 3. Results

This section presents the empirical findings from the inductive (derived from the data without pre-imposed theoretical frameworks) RTS [79,80,81,82,83,84] of eight peer-led group sessions conducted by autistic young adults between April and May 2025 within an SVE. Sessions were autonomously facilitated by participants, who collectively selected discussion topics beforehand. All sessions were recorded, transcribed verbatim, and iteratively coded to develop session-level themes. Session characteristics (number of participants, gender group composition, topic, and duration were documented) varied and are reported in Appendix A.

Rather than presenting findings on a session-by-session basis, our analysis focused on identifying cross-session themes of meaning while retaining heterogeneity in how experiences were described and situated by the autistic young adults. Themes were developed through systematic coding and theme generation of the transcribed material, followed by iterative clustering and refinement across multiple analytic rounds.

### 3.1. Topics: Autistic Young Adults’ Discussion Priorities

Across the eight sessions (see: Appendix A), participants selected the following topics: (1) friendships and neurodiversity; (2) self-perception and disability; (3) challenges in social interaction; (4) societal change and representation; (5) fears, aspirations, and future imaginaries; (6) diagnosis and stimming; (7) spirituality; (8) education system/schooling experiences, including desired changes. These topics structured the sessions and informed the content of the themes reported below.

#### 3.1.1. Cross-Session Themes (Thematic Clustering)

Across eight peer-led discussion sessions, participants described autistic life in relation to social systems organized around NT norms. Themes were developed reflexively through iterative engagement with the dataset, attending both to individual contributions (i.e., participants’ distinct experiences, perspectives, and positions) and to collective meaning-making, through which these contributions were discussed, compared, negotiated, and, at times, converged into shared understandings during group interaction. In this sense, the analysis did not treat contributions as isolated individual accounts, nor did it reduce discussion to a single group consensus, but considered how meanings emerged through interaction as participants responded to and built on one another’s accounts.

Four interrelated macrothemes were developed, each capturing shared patterns alongside heterogeneity within-themes (i.e., participants varied in how, when, and where experiences were felt as disabling). The macrothemes are interrelated in that they were not observed as separate or independent domains of experience, but as analytically distinct aspects of the same situations and narratives. Thus, the macrothemes are linked through co-occurrence and mutual influence within participants’ lived experiences, rather than through a linear, causal, or developmental sequence.

In fact, across the dataset, participants move from making sense of self (Macrotheme 1), to negotiating relational risk and desire (Macrotheme 2), to revealing the hidden labor required in socio-relational contexts (Macrotheme 3), to locating misfit in institutions and articulating actionable futures oriented toward access, dignity, and shared benefit (Macrotheme 4).

##### Macrotheme 1—(Finally) ‘Becoming Autistic’: Diagnosis, Identity, and Self-Definition

Autistic young adults described autism primarily as difference rather than defect, while noting that difficulty, or disability, emerges in environments that demand sustained NT interactional performance. Several participants contrasted domains in which they felt highly capable with others in which they experienced distress:

“I agree, in some work-tasks I feel like I have superpowers, while in others I feel disabled […] like when I have computer-related tasks I can learn new softwares real-quickly and am usually ahead of all my neurotypical colleagues, but when I am working frontdesk—I just can’t!”

Diagnosis was frequently framed as an interpretive resource that enabled retrospective sense-making. Participants emphasized that diagnosis did not “create” difference but provided language to understand long-standing experiences of being perceived as “not normal”, “different from everyone else”, or “crazy”. Timing of diagnosis (early vs. late) was described as less important than how diagnosis supported self-understanding and self-awareness, self-advocacy, and communication with others.

Language choices around diagnostic labels were treated as ethically significant. While some participants used the term *Asperger*, most rejected it as outdated and expressed concern about labels “replacing genuine understanding of the individual”.

Participants also challenged stereotypes portraying autistic people as lacking empathy or emotional capacity. Emotional experience was described as intense but often expressed in ways misread by NT observers: “It’s not that neurodivergent people don’t know how to communicate, they just communicate differently”.

Alongside diagnostic narratives, participants described stimming and focused interests as affirmative and embodied routes into self-understanding and regulation. Stimming was framed as multifunctional (e.g., regulating sensory overload, supporting concentration, and expressing excitement) and as closely tied to reduced self-surveillance and increased permission to be oneself. Interests were framed as central to identity, continuity, and wellbeing, and often as relational bridges. Spirituality was discussed heterogeneously and was generally framed as a moral orientation (“doing good”) and community contribution rather than doctrine: “I am a believer, regardless, I try to do good in my community; I see this as the true meaning of spirituality”.

##### Macrotheme 2—Relating on the Edge of Belonging: Friendships, Family, and Social Pain

Participants consistently described the social world as being desired and risky (i.e., friendship building and maintenance challenges, rejection, bullying, family misunderstandings, and ND-ND ease versus ND-NT friction). While many recognized themselves in the stereotype “autistic people struggle with friendships”, difficulty was framed as context-dependent rather than as lack of social motivation. Participants reported vulnerability to being misread and excluded or penalized quickly, particularly in new social groups. Distinctions were drawn between emotionally meaningful “deep friendships” and more instrumental and superficial connections described as “convenience companionships”. Many described becoming more selective with age and preferring solitude over relationships that felt inauthentic, judgmental, or emotionally unsafe. Conversation itself was often described as cognitively demanding: volunteers reported uncertainty about when to join a discussion, how much detail was appropriate, and whether detailed responses would be welcomed or penalised.

Interactions with other ND individuals were frequently described as easier, safer (“we reassure each other”), and less judgmental, with divergent communication styles being accepted (e.g., not maintaining eye contact, shifting posture, walking or stimming while listening to someone speak). In contrast, NT contexts were described as requiring closer adherence to implicit social scripts and greater monitoring of conversational behavior (i.e., pacing and “appropriate” emotional expression).

“[…] it’s funny because it’s the psychology department of the university […] my [psychology] classmates should be a bit less judgmental [towards autism/autistic characteristics].”

Family relationships were often described as painful sites of misunderstanding and dismissal. Experiences of bullying and social exclusion—particularly during schooling—were reported as formative, with ongoing effects such as heightened vigilance, social anxiety, fear of rejection, and rumination.

“I sometimes still feel like an alien”

##### Macrotheme 3—The Hidden Labor and Costs of Adaptation and Participation: Masking, Monitoring, and Strategic Withdrawal

Participants described social participation in NT-oriented settings as requiring sustained, and often invisible, cognitive, emotional, and sensory labor. This included rehearsing conversations (“I practiced in front of the mirror”), actively monitoring timing and turn-taking, managing disclosure (‘impression management’), and adapting to sensory environments. 

Masking was described as ambivalent: protective in some contexts (i.e., avoiding overt stigma), but cumulatively exhausting and, for several participants, associated with burnout and loss of spontaneity, creativity, and embodied ease (“as if I have chains on when surrounded by neurotypical people”).

“They [NTs] have the possibility to adapt whereas autistic people pay the price in burnout if we do all the adapting to systems that aren’t at all in alignment with neurodivergent ways”

Strategic withdrawal and boundary-setting were described as protective responses to repeated and persistent misattunement (“If I can avoid it, I avoid it, because I don’t want to feel bad anymore”). Solitude was framed variably—as regulation and safety for some, and as an unwanted consequence of exclusion for others. Across accounts, participants emphasized that perceived overall capacity fluctuated according to environmental contexts (i.e., environments that are predictable, structured, and sensory-manageable were associated with competence and agency, whereas environments that amplify sensory and social demands were associated with distress and functional difficulty or challenges).

##### Macrotheme 4—Misfitting Systems and Livable Futures: Education, Work, and Support Redesign

Participants located many disabling experiences within institutional structures or design rather than autism itself.

Educational contexts were described as privileging implicit inference (“having to guess something that wasn’t written”) and rote repetition, while moralizing fluctuations in performance as lack of effort (“if you don’t apply yourself, it’s treated as your fault, rather than anyone asking what else might be happening at home or in your head”). Autistic young adults reported that variability (e.g., being “ahead” at times and unable to study at others) was rarely interpreted as a signal of contextual stress, burnout, or unmet needs. Sensory conditions in the classroom (i.e., noise, crowding, and unpredictable classroom input) were frequently linked to shutdown, emotional distress, and withdrawal. Support services were described as inconsistent, standardized (“[…] speaking ‘from behind the scenes’ as a [autistic] teacher, I observed that those [professionals/teachers] who deal with diversity and inclusion often don’t really want to understand and instead try to ‘put a patch’ on the problem with generic solutions that don’t fit the individual […]”) or oriented toward compliance rather than enabling authentic participation (“support teachers focused too much on the social side, it was suffocating […] ‘go play with the other kids’ […]”). Several participants reported refusing support not because it was unnecessary, but because it was experienced as intrusive, misaligned, and, sometimes, harmful.

“[…] demanding, pressing requests […] ‘go do this, go do that’, ‘go and stay with your classmates’ […] it can be a bit suffocating […] I said ‘no more’, I no longer wanted educators or support teachers”

“she [the teacher] was supporting me so much, it was helpful, but it made me feel different” (autistic volunteer shares how they felt treated as an exception rather than the educational system simply recognizing every student has different needs)

Workplaces were similarly described as contexts in which ability, skill, and potential coexisted with difficulty and ‘dis-ability’ depending on environmental design, role demands, and accommodations. Participants shared that they wish employers and colleagues could: “see autistic employees as people who can bring value to the company through their work, abilities, and different ways of seeing reality”.

Participants articulated features of more livable environments (e.g., permission to use headphones, quiet rooms, predictable meetings, fewer unplanned interruptions, and flexibility in breaks and movement) and emphasized that such adjustments could benefit everyone, beyond autistic employees.

Professional support systems (clinical, psychological, and educational) were frequently described as “manual-based” and insufficiently responsive to the (autistic) person in front of them (“[…] I was seeing a school therapist for six years who knew nothing about autism and had no idea how to help with autistic-specific needs”). Participants called for support grounded in listening, practical competence, and—recurrently—greater representation of ND professionals.

Future-oriented discussions emphasized structural and cultural change rather than individual correction, including normalization of neurodivergence (“value different cognitive styles”; “allow everyone to learn according to their own modalities”) and redesign of systems (schools, workplaces, services, and the media) to reduce stigma, interpretive burden, and the need to mask.

### 3.2. Observational Notes Collected

In addition to transcript analysis, the research team produced observational memos to characterize group processes in the SVE and to inform the methodological feasibility of peer-led autistic inquiry in VR. Across sessions, participants used multimodal communication—voice, emoji reactions, avatar gestures and movements, and chatbox—as interactional feedback and as a means of participating without interrupting speakers. Overlapping starts in turn-taking occurred periodically and were typically repaired through apologies and yielding the floor, indicating active monitoring of conversational flow despite platform constraints.

Autistic young adults also demonstrated informal, distributed facilitation: at different points, the autistic volunteers assumed mediating roles (e.g., managing hand-raising, clarifying platform mechanics, and relaying time-limited chat messages to ensure accessibility and inclusion). Engagement was not reducible to speech alone: some participants contributed minimally verbally but displayed attentiveness through avatar orientation, emoji responses, or brief chatbox participation. Finally, participants occasionally expressed sensitivity to environmental affordances and coherence within VR (e.g., preferring worlds with concrete seating objects) and to disruptions introduced by world transitions, underscoring the relevance of stability and accessibility (including cross-device compatibility) in designing VR-based group research settings.

## 4. Discussion

### 4.1. Private-World VRChat Sessions as a Research Method

During the Community Partners meetings, autistic volunteers described the research experience as positive and meaningful, characterizing the sessions as “fun” and “positively lived”. The sessions were framed as opportunities to come together, share lived experiences, and, as one participant expressed: “throw our worries, [negative] past experiences, into a metaphorical fire and unite as a group”. Participants consistently emphasized mutual respect and non-judgement, describing the environment as a “safe place” that supported sharing and a sense of comfort grounded in shared expectations (“rules”) around “living and interacting well” within the group. Experiences of safety, shared expectations, and non-judgement align with prior qualitative work showing that autistic people often find structured, predictable, and autism-informed social contexts more accessible and less threatening than conventional face-to-face interactions [22,23,40].

Both participants’ feedback and observational data indicate that the VR-based setting supported engagement and self-expression without being experienced as coercive. Several participants described the virtual environment as gently encouraging them to “put ourselves out there” and open up. At the same time, some participants reported challenges related to concentration, limited familiarity with virtual environments, or minor technical issues (e.g., audio or volume adjustments). Despite these challenges, VRChat was generally perceived as intuitive, and the overall experience was evaluated as enjoyable and worthwhile. This suggests that the present setting supported engagement without reproducing the longstanding concerns that autism-related research can inadvertently promote coercive or masking-based participation [36,37,50].

Participants also described the sessions as spaces for emotional expression and community-building. Hearing others articulate shared difficulties within the same environment contributed to a sense of connection and “a bit of community”. When asked to summarize their experience using a single word of their choice, participants most frequently referred to “safe place”, “respect”, “inclusion”, and “belonging”. Observational data further supported these accounts, documenting active engagement, empathy, humor, collaborative problem-solving, and sustained efforts to maintain group cohesion. Reports of connection and emerging community resonate with evidence that autistic-autistic (ND-ND) interaction is often experienced as more comfortable and mutually intelligible, supporting belonging and shared understanding [17,85,86].

Importantly, engagement extended beyond the formal research sessions. Autistic partners continued to communicate via Discord, met independently in VRChat, and in some cases reported meeting in person or planning to do so in the near future. This sustained engagement suggests that the research environment supported meaningful social connections rather than purely task-oriented participation. Moreover, sustained engagement beyond formal research context echoes findings that autistic people value ongoing social connection—both close ties and more informal, low-stakes interactions—as central to well-being [87,88] and supports conceptualizations of VR as a connective or transitional space rather than a social substitute for in-real-life [40].

Taken together, these findings suggest that private-world VRChat sessions can function as a participant-centered research method that supports both ethical engagement and methodological quality. The setting allowed participants to regulate how, how much, and when they engaged, while still enabling in-depth discussion and shared meaning-making. In this exploratory study, prioritizing participants’ comfort, agency, and relational safety did not reduce the richness of the data; instead, it supported sustained, focused exchanges and ecologically grounded insights. More broadly, these findings highlight the potential of SVEs for autism research when designed and facilitated with participants’ needs treated as non-negotiable [63,89].

These findings contribute to growing evidence that digital- and VR-mediated spaces may function as enabling contexts for autistic social connection [22,23,40], partly because they allow participants to negotiate social interaction on their own terms—through multimodal cues, user-controlled pacing, and concrete platform affordances (e.g., spatialized audio, adjustable proximity, muting, and avatar-mediated presence). SVEs seem to provide autistic adults with a setting that can support flexible engagement, reduce interactional ambiguity, and foster shared understanding. Importantly, prior research cautions against positioning such environments as replacements for in-person interaction; instead, they appear most valuable as situational supports that reduce social threat while preserving agency and choice [40]. The present findings extend this literature by demonstrating how these affordances can also support ethical, participant-centered qualitative research practice.

### 4.2. Autistic Point of View

From a current literature perspective, the five macrothemes generated through our cluster analysis can be interpreted abductively through several well-established frameworks in contemporary autism scholarship. The Double Empathy Problem [5,6,7,8,68] helps explain how bidirectional misattunement between autistic and non-autistic people is often misinterpreted as an individual ‘deficit’, while more recent work has proposed a “triple empathy problem” in professional contexts (e.g., healthcare), where misunderstanding may be further compounded by institutional and cultural divides between professionals and non-professionals [90]. The results are also consistent with minority stress theory [91,92], accounts of chronic social strain (e.g., autism stigma [36]), analyses of ableism [93], and broader critiques of how power asymmetries can marginalize neuro-minorities through environments that remain inadequately or insufficiently accommodating or understanding (e.g., NT social norms embedded as default) [70,94,95]. Moreover, to explain how these socio-contextual dynamics are processed internally by the autistic person, we propose to look at their effects on autistic identity formation and daily coping. Specifically, we propose an ego–psychological [95] reading as a complementary lens for understanding how contemporary conditions of autism stigma and NT normativity shape self-experience in practice.

From an ego-psychological perspective [95], the macrothemes describe repeated encounters in which autistic participants must negotiate the integrity of the Self (i.e., protecting continuity and negotiating self-coherence, dignity, and legitimacy under conditions of misrecognition). When everyday interaction repeatedly requires or demands ongoing translation into NT norms and/or expectations, autistic individuals may rely on coping strategies that might preserve social belonging but tax the Self: heightened self-monitoring, strategic inhibition, self-silencing, and ‘passing’ practices (often discussed in autism research as camouflaging/masking [33]). In ego-psychological terms, these strategies can be understood as adaptations serving self-protective functions (e.g., reducing interpersonal threat, preventing shame, and avoiding conflict). Yet, aligned with the current state-of-the-art, they can also carry cumulative costs, including autistic ego-depletion, exhaustion or burnout [77], identity strain or diffusion (‘Who am I when I am not performing?’), and a persistent sense of conditional acceptance. This framing helps connect macro-level processes (e.g., ableism [51,93], stigma [36,94], and minority stress [91,92]) to micro-level phenomenology, highlighting how structural mismatch is metabolized as lived experience through a continuous demand to regulate visibility, legitimacy, and emotional safety. This lens also clarifies how relational environments shape the conditions under which authentic self-expression becomes possible. Across macrothemes, participants suggested that well-being is not the outcome of individual social skill acquisition as much as it is a function of entering or joining contexts that legitimize directness, depth, and support mutual recognition, predictable interactional expectations, and non-punitive interpretations of difference.

As witnessed through this study, experiencing what autistic participants described as a ‘safe (research) environment’, reduced their need for defensive self-management, enabling more expansive forms of engagement, including curiosity, humor, playfulness, vulnerability, and sustained collaborative meaning-making. The implication is that what is often labelled ‘impairment’ or ‘deficit’ may, in everyday life, reflect a mismatch between the person and the social ecology. This is a point already central to empathy problem interpretations, but—with our proposed interpretation—we are extending it to identity consequences: chronic mismatch does not only hinder interaction; it can shape Self-relations through repeated experiences of being perceived as “too much”, “not enough”, or “wrong”. Repeated negative social experiences – often described in the literature as “polyvictimization” [96] – may help explain the elevated rates of Post-Traumatic Stress Disorder reported among autistic adults [97]. When such experiences are internalized and compounded by marginalization and unmet support needs [98], they may also contribute to the disproportionately high prevalence of suicidal thoughts and behaviors observed in this population [32]. Together with the present findings, recent research [32,97,98] underscores the urgency of rethinking how autistic people are seen, understood, treated, and supported. In line with this, our results support ongoing calls to move beyond deficit-based framings of neurodivergence and toward neurodiversity-affirming models that recognize autistic ways of communicating and relating as legitimate variations rather than errors to be corrected or eliminated [91].

Finally, it is clear why neurodiversity-affirming, autism- and trauma-informed research practices in research matter beyond ethical considerations alone. Disclosure and participation are shaped by prior experiences of misunderstanding and social risk; research encounters are never neutral. Methods that reduce threat, support autonomy, and allow participant-led pacing can alter what becomes speakable and how meaning-making unfolds. In this sense, the macrothemes document not only autistic experiences in the world, but also the conditions under which autistic individuals can maintain self-coherence, relational safety, and agency while engaging with others—an insight with direct relevance for both methodological and clinical debates in autism research.

## 5. Conclusions

### 5.1. Originality and Contributions to the Field

The originality of this study lies in considering social interaction as a relational and situational (contextual) achievement, rather than as an individual competence or deficit [32,86,99]. Moreover, social VR is conceptualized not as a tool for remediating autistic behavior or training normative social skills, but as an alternative social ecology in which autistic people can collectively share experiences, discuss priorities, and negotiate interpretations on their own terms [32,86,99]. This positioning responds directly to calls for neurodiversity-affirming and participatory autism research that prioritizes ecological validity and the redistribution of interpretive authority [50].

In doing so, this study contributes beyond existing VR and online-interaction research by providing exploratory insight into how autistic young adults collectively make sense of sociality when environmental demands are altered, instead of individual behavior being targeted. In particular, it contributes to (a) the aforementioned neurodiversity-affirming reconceptualization of socio-cognitive and affective challenges as context-sensitive mismatches rather than intrinsic deficits; (b) insight into potential protective processes (e.g., greater autonomy in managing sensory and cognitive demands; reduced pressure to mask; and avatar-mediated control over self-presentation) that may support well-being; and (c) methodological advancement in the use of immersive SVEs as novel, feasible, and acceptable sites for participatory, peer-led qualitative autism research. 

Overall, this study extends prior qualitative research on autistic experiences in digital and virtual environments; complements participatory autism research by operationalizing shared interpretive authority through virtual participatory practices; and diverges from intervention-oriented VR research by reframing immersive technologies away from normative remediation goals. More broadly, the findings support a shift from individual-focused intervention toward context redesign, highlighting the methodological and conceptual value of SVEs as ethical, participant-centred, and ecologically meaningful spaces for autism research [88].

### 5.2. Limitations and Future Directions

Several limitations should be acknowledged. First, the sample size was small, as is typical for exploratory, qualitative research. Accordingly, analytic claims are based on patterns of meaning-making that recurred across sessions rather than in prevalence or distribution, and the findings are not intended to be generalizable to the autistic population at large. In fact, given the well-recognized heterogeneity of autism spectrum conditions, the findings should be interpreted as reflecting the experiences and perspectives of a formally diagnosed, low-support-needs group participating in a specific research context, rather than representative of the entire autistic population. The study was not designed to capture the full diversity of autistic presentations. We have therefore intentionally avoided population-level claims and frame our contributions as exploratory and theory-informing, aimed at advancing conceptual understandings and methodological insight rather than providing definitive or population-level conclusions.

Second, although the age range (18–38 y.o.) and gender distribution of the participants reflects the natural composition of the participating group, the study was not designed to examine age-related and gender-related variations. Age and gender were treated as demographic characteristics rather than analytic variables, and the sample does not support age-stratified or gender comparisons. While participants occasionally reflected on differences between earlier and later adulthood, as well as gendered differences in social treatment, these reflections were analyzed as experiential, shared meaning-making content rather than developmental trajectories.

Third, information regarding time since diagnosis was not systematically collected or analyzed, in accordance with ethical practices such as data minimization principles. Although all participants voluntarily shared this information (it was available to the research team if necessary), it was not formally recorded for this specific exploratory study, as it was not required for its aims. Such disclosures were treated as contextual, collective information rather than as variables for comparison. We acknowledge that differences in diagnostic history may relate to self-understanding, access to support, and coping strategies and could have shaped participants’ experiences in ways not disentangled here. We identify this as an important direction for future research, particularly for studies examining how diagnostic pathways intersect with participation, self-advocacy, and identity development.

Furthermore, although prior familiarity with VRChat and immersive virtual reality was controlled through eligibility criteria, broader differences in digital literacy, comfort with online interaction, and/or prior engagement with digital social spaces, may have influenced how participants navigated and participated in the social virtual environments. For example, such differences could have shaped participants’ ease in using platform features, their willingness to engage multimodally, or their confidence in initiating interaction. Although information regarding participants’ digital practices and preferences was collected as part of the broader research project, these data were not incorporated into the present analysis, as they were not central to the exploratory aims of this study and would not have meaningfully informed the inductive, participatory-focused analytic strategy adopted. Accordingly, analytic claims are grounded in shared interactional and experiential patterns observed across sessions rather than individual task performance or technological proficiency. Future research could benefit from explicitly examining how digital familiarity intersects with participation, accessibility, and meaning-making in immersive social environments.

Finally, a key future direction concerns the role of artificial intelligence (AI) within social VR environments used for research. AI-mediated moderation, accessibility, summarization, immediate technical support tools (i.e., real-time captioning and AI-buddy to remind autistic-users of interoceptive cues, like thirst and hunger when exploring virtual worlds for longer periods) could support safer participation and reduce barriers for some autistic individuals, particularly when attention, sensory load, or real-time language processing fluctuate. At the same time, AI introduces new risks—especially around privacy, consent, and data governance that warrant explicit attention (i.e., surveillance, unintended recording, biometric inference, and secondary data use)—that need to be addressed in order to ensure that these environments remain safe and trustworthy.

## Data Availability

The dataset generated and analysed may be made available by the corresponding author upon reasonable request and subject to ethical approval.

## Abbreviations

The following abbreviations might have been used throughout this manuscript:

AI	Artificial Intelligence
CBPR	Community-Based Participatory Research
DSM-5-TR	*Diagnostic and Statistical Manual of Mental Disorders, Fifth Edition, Text Revision*
ND	Neurodivergent
NT(s)	Neurotypical(s) (or allistic)
RTA	Reflexive Thematic Analysis
SVE(s)	Social Virtual Environment(s)
VR	Virtual Reality
AFAB	Assigned Female At Birth
AMAB	Assigned Male At Birth

## Appendix A

This appendix provides a structured overview of the nine sessions comprising Phase Three of our intervention design. The intervention was developed based on findings from prior research aimed at designing an effective digital and virtual intervention for autistic young adults. Specifically, the intervention design was informed by (a) an exploratory qualitative study of publicly available self-generated content by autistic users, focusing on personal experiences, perceptions, and feedback on social virtual environments [21]; (b) a cross-cultural study including focus groups with Italian autistic and allistic (or NT) autism professionals and in-depth interviews with ND professionals from the United States and Australia [22]; and (c) interviews and focus groups with autistic young adult volunteers. Throughout the intervention delivery, the design was iteratively refined in response to ongoing observations and constructive feedback provided by participating autistic young adults.

The table below (Table A1) presents a detailed timeline including session dates, duration, attendance, concise summaries of each session’s participant-selected topic, and the total number of themes created per session through an RTA. Observations were documented in reflexive memos drafted independently by the research team and subsequently discussed. More detailed process documentation may be available upon request.

**Table A1:** Session Timeline and Overview (N = 9 VRChat sessions).

Date of Session	Discussion Duration	Total Number of Participants	AFAB	AMAB	Topic (Translated from Italian to English)	Total
						Main Themes Developed (RTA)
6 April 2025	~133 min	9	4	5	N/A (Transition to and familiarization with the group)	N/A
	(~2 h 13 m)					
13 April 2025	~70 min	11	6	5	Neurodiversity, with a focus on friendships	7
	(~1 h 10 m)					
18 April 2025	~80 min	8	4	4	The perception of one’s neurotype, with a focus on disability	7
	(~1 h 20 m)					
27 April 2025	~60 min	10	4	6	What are the biggest challenges faced when interacting with others?	4
	(~1 h)					
4 May 2025	~95 min	10	5	5	What would you like to see in today’s society to help integrate ND people into the collective imagination?	5
	(~1 h 35 m)					
9 May 2025	~53 min	6	3	3	What are the greatest fears associated with being autistic, and what hopes and aspirations do you hold for the future?	2
16 May 2025	~47 min	6	3	3	How did you experience receiving an autism diagnosis? What types of stimming do you prefer?	4
25 May 2025	~58 min	9	3	6	Session 6 topics [continue] + addition of the topic “Spirituality”	4
30 May 2025	~100 min	7	4	3	Didactics: how did you experience school and teaching/learning as autistic individuals – whether you were diagnosed early or later in adulthood? In what ways did this affect your educational journey, positively or negatively? And if you had been given the chance, is there anything you would have changed?	3
	(~1 h 40 m)					
